# Impact of Multienzymes Dose Supplemented Continuously or Intermittently in Drinking Water on Growth Performance, Nutrient Digestibility, and Blood Constituents of Broiler Chickens

**DOI:** 10.3390/ani10030375

**Published:** 2020-02-26

**Authors:** Youssef Attia, Mahmoud El-kelawy, Mohammed Al-Harthi, Ali El-Shafey

**Affiliations:** 1 Arid Land Agriculture Department, Faculty of Meteorology, Environment and Arid Land Agriculture, King Abdulaziz University, P.O. Box 80208, Jeddah 21589, Saudi Arabia; 2 Department of Poultry Production, Faculty of Agriculture New Valley University, El-Karga- 72511, Egypt; M.Elkelawy@gmail.com; 3 Animal and Poultry Production Department, Faculty of Agriculture, Damanhour University, Damanhour 22516, Egypt; alielshafey2015@gmail.com

**Keywords:** broilers, multienzymes, administration method, enzyme dose, performance, blood constituents

## Abstract

**Simple Summary:**

Supplementing enzymes in diet can improve animal performance, carcass traits, physiological status, and reduce cost of feeding due to improving feed utilization. Enzyme supplementation in water is simple to apply, disseminates, and the contact with the substrates is faster. In addition, water supplementation may lessen the negative effects of aggressive heat exposure on enzyme activities when the pelleting temperature exceeds 85 °C and can replace expensive post-pelleting spraying systems. We investigated the effect of different doses of enzymes supplemented in water either continuously or intermittently on growth performance, digestibility of nutrients, and blood profiles. Results indicated that intermittent supplementation of enzymes at 1 mL/L drinking water and continuous supplementation at 0.5 mL/L drinking water can be investigated in further experiments as a tool to improve broiler performance and European production index.

**Abstract:**

The aim of this work was to study the continuous or intermittent impact of a multienzyme supplement on growth performance, nutrient digestibility, and blood metabolites of broilers, and to evaluate production index of dietary supplementation. A total of 315 unsexed day-old Arbor Acres broiler chicks were randomly distributed to seven treatments groups, keeping initial body weights similar, in 35-floor pens (replicates) of nine chicks per replicate (pen) and five experimental units per treatment. All experimental groups were fed the same basal diet and administered seven multienzyme treatments: the 1st group (control) did not receive any enzyme supplementation; the 2nd, 3rd, and 4th groups were administered multienzymes at 0.5, 1.0, and 1.5 mL/L drinking water, respectively. Each enzyme supplemented-group was divided into two subgroups, with additives being applied either continuously (24 h/day) or intermittently (12 h/day) from 1 to 35 days old. Regardless of administration method, multienzyme supplements at 1.0 mL/L water along with a corn-soybean meal diet increased the body weight gain (BWG) by 7.8% compared to 0.5 mL/L water during days 1–21 of age. In addition, 1.5 mL/L water significantly improved BWG by 5.1% of broilers compared to 0.5 mL/L water during days 1–35 of age. Enzyme supplementation at 1.5 mL/L water significantly enhanced feed conversion ratio (FCR) by 4.3% during days 1–21 of age, and FCR by 5.2% and European production index (EPI) by 10.4% during days 1–35 of age compared to the group on 0.5 mL/L water. For the whole period, there were improvements of beneficial consideration in BWG (4.0%), FCR (4.0%), and European production index (8.2%) due to continuous multienzyme supplementation at 0.5 mL/L water compared to the same dose added intermittently. A similar trend was observed due to intermittent multienzymes at 1 mL/L drinking water that resulted in increased BWG by (6.4%) and improved FCR by (6.7%) and EPI by (12.7%). Intermittent administration significantly increased feed intake of broilers during 22–35 days of age compared to continuous supplementation. Multienzymes at different doses did not significantly affect the digestibility of nutrients, blood serum biochemical constituent, inner body organs, and markers of functions of liver and renal organs. In conclusion, the highest BWG and the best FCR and EPI for the whole period were from broilers given continuous 1 and 1.5 mL/L drinking water or intermittent multienzyme supplementation at 1.5 mL/L drinking water. Furthermore, intermittent supplementation of enzymes at 1 mL/L drinking water and continuous supplementation at 0.5 mL/L drinking water can be investigated in further experiments as a tool to improve broiler growth performance and economic traits and to decrease the cost of enzyme application.

## 1. Introduction

Diet composition is a key factor affecting the response to enzyme supplementation in poultry [1,2,3]. Vegetable based-diets contain anti-nutritive substances such non-starch polysaccharides (NSP), tannins, trypsin inhibitor, and phytic acid that negatively influence animal performance, digestibility of nutrients, environment, and decrease gut health and feed utilization [4,5,6,7].

Enzymes are commonly employed to decrease anti-nutritional substances and to improve animal performance [8,9,10,11]. Multienzymes containing carbohydrases and phytase were found to enhance the utilization of energy, protein, and minerals by chickens [12,13,14], suggesting that higher amounts of alternative feedstuffs could be used in the presence of enzymes [15]. Rye, wheat, and barley grains, which contain high levels of soluble-NSP, particularly arabinoxylans (pentosans) pectin, and β-glucans may reduce the rate of gut emptying and affect small intestinal transit time, block fats from digestion and thus absorption, therefore, cannot be incorporated into chickens’ diets at high concentrations unless exogenous enzymes are adequately applied. It has been well documented that the high level of soluble NSP in rye increases digesta viscosity and the stickiness of droppings, which results in poor poultry performance [16,17]. 

Evidently, enzyme additions to corn, wheat, barley, and rye diets have been shown to significantly improve body weight gain (BWG) and feed conversion ratio (FCR) in broilers [11,16,18]. Generally, these enzymes are hypothesized to work in two steps, described as an ileal phase and a cecal phase [19,20]. During the ileal phase, enzymes remove fermentable substrates. During the cecal phase, degradation products of sugars, such as xylose and xylo-oligomers, are fermented by cecal bacteria, stimulating the production of volatile fatty acids (VFA) and the growth of specific beneficial bacteria [6]. In the process of depolymerization various polysaccharides in the diet, enzymes may produce manno-, galacto-, gluco-, or xylo-oligomers, which are similar to prebiotics and which may facilitate the proliferation of health-promoting bacteria such as *Lactobacillus* and Bifidobacterium [21]. Cellulase is a viscosity-reducing enzyme and is a group of enzymes that hydrolyze cellulose or β-(1,4)-glucan [2,14]. Protease enhanced degradation of soybean meal protein in the gut notably, and the mode of action of protease are wholly allied with the digestibility [14]. This observation has been evidenced by a significant increase in growth and an improvement in gut health and FCR when broilers were fed corn-based diets supplemented with enzymes [20,22,23].

In the available literature, there is a large body of results on feed supplementation with enzymes on broiler performance in contrast to the use of enzymes in drinking water. However, enzyme supplementation in water is more simple to apply, disseminate, and contact with the substrates is faster [24]. In addition, water supplementation of enzymes may lessen the harmful effects of aggressive heat exposure on enzyme activities when the pelleting temperature exceeds 85 °C and can replace expensive post-pelleting sparing systems [25]. Enzyme supplementation in drinking water significantly increased the body weight of broilers during different periods of growth, from 14 to 35 days of age, from 20 to 41 days, and from 20 to 40 days of age [26,27,28,29]. In addition, broilers that received multienzymes through drinking water recorded the highest weekly weight gain when compared to those given enzymes through the feed, and both groups had higher growth than unsupplemented controls [24]. Thus multienzymes through drinking water at 0.5 g/L had a positive and growth-boosting effect in broiler chickens. Recently, feed additives may be used intermittently and resulted in similar growth performance and more economic benefits than continuous supplementation [30]. 

The enzymes’ influence on blood constituents may shed light on the impact of enzymes on metabolic processes. However, contradictory results were found in the literature. Multienzyme supplementations at 0.5–1 mL/L water significantly decreased alanine aminotransaminase (ALT) and aspartate aminotransaminase (AST), showing an improvement in liver leakage markers [26,27,28,29]. On the other hand, plasma total protein was higher in enzyme supplemented-groups than of controls, but albumin, globulin and albumin/globulin ratio, ALT, and AST were not affected [13]. Enzymes also increased total protein, albumin, globulin and albumin/globulin ratio compared to controls, but liver leakage markers (AST and ALT) were not affected by enzymes [3,31]. However, an enzyme cocktail did not influence the plasma biochemical constituents or the indices of the liver function of broilers [23,32]. 

Hence, this study aimed to evaluate the effects of multienzymes given in water, either continuously or intermittently, on productive performance, nutrient digestibility, carcass characteristics, and indices of the liver and renal functions of broiler chicks from 1 to 35 days of age.

## 2. Materials and Methods 

All procedures were approved by the Deanship of Scientific Research (DSR), King Abdulaziz University under proposal number D-182-155-1440 H, that recommends animal rights, welfare and minimal stress, and did not cause any harm or suffering to animals according to the Royal Decree number M59 in 14/9/1431H.

### 2.1. Chick, Supplement, Design, and Husbandry

A total of 315 unsexed day-old Arbor Acres broiler chicks were obtained from the Cairo hatchery, wing banded, and randomly distributed to seven treatment groups, keeping initial body weight similar, in 35-floor pens (experimental unit) of nine chickens per pen and five replicates per treatment. All experimental groups were fed the same basal diet (Table 1) and were given seven multienzymes (Caplix^®^ is a product of Vetoquinol India; Caplix^®^ contains multienzymes 100,000,000 U/L cellulase, 1,500,000 U/L xylanase, 250,000 U/L amylase, and 400,000 U/L protease) treatments as follows: the 1st group (control) did not receive enzyme supplementations; the 2nd, 3rd, and 4th groups were given multienzymes (Caplix®) at 0.5, 1.0, and 1.5 mL/L drinking water, respectively. Each supplemented group was divided into two subgroups, in which the additives were administrated continuously (24 h/day) or intermittently (12 h/day), respectively, during the 1st through to the 35th day of age. The enzyme was added to water tank daily for those given continuous access, while it was added from 8 am to 8 pm for those intermittently supplemented. The water system (tank, tube, and cups) was flushed with clean water in-between enzyme applications to avoid the residual of enzymes in the water system. The water used was tap water recommended for human consumption in Egypt. Water intake was not determined herein due to limited research facility. 

### 2.2. Housing and Husbandry

Chicks were housed in floor brooders in a semi-opened house. Their light schedule was 23 h light up to 21 days of age, followed by 20 h of light until slaughter. The average outdoor minimum and maximum temperature and relative humidity during the experimental period were 22.3 and 25.6 °C and 55.4% and 58.3%, respectively. The housing temperature was 32 °C during the 1st week and declined gradually by 2 °C each week and was then stabilized at 28 °C until slaughter. Chicks were vaccinated against the most common diseases, such as Newcastle disease (ND), infectious bursal disease (IBD), and infectious bronchitis (IB), under veterinary care. The experimental diets were formulated to meet the nutrient requirements of broiler chickens [33]. The ingredients and the calculated [33] and determined [34] composition of the experimental basal diets fed during the two phases of broiler production (starter and growing periods) are shown in Table 1. Crumble feed and water were given ad libitum.

### 2.3. Response Traits

Broilers in each replicate were weighed (g) at 1, 21, and 35 d of age, and the BWG (g/chick) was calculated. Feed intake was recorded for each replicate (g/chick) and thereby FCR (g feed/g gain), and survival rate (SR, 100 - mortality rate) during 1–21, 22–35, and 1–35 d of age were calculated. The European production efficiency index was calculated [8].

The apparent digestibility of crude protein (CP), ether extract (EE), crude fibre (CF), and ash was performed using five replicates of three males housed in metabolic metal cages per treatment in a separate trial [8]. The excreta of the total tract were collected using the total tract apparent digestibility method during 36–40 days of age [8]. To test the apparent nutrient digestibility, the experimental period was 5 days: 2 days of adaptation and 3 days of the collection period in which non-marked excreta during days 38 and 40 of age was discarded. During the collection period, feed intake and voided excreta were recorded daily. To identify the excreta derived from the tested diets, 1% of ferric oxide was supplemented to the tested diet at the 1st and last day of collection, 38 and 40 days of age, respectively [35]. Hence, non-marked excreta of the 1st and 3rd days of collection period were discarded. The excreta was collected from each replicate, cleaned from feed, feather, and scales and pooled for the three days, dried in a force ventilated oven, stored in glass jars, and kept for further analyses. Nitrogen as a faecal nitrogen was determined after separation of urinary nitrogen according to [36] while EE, CF, and the ash content of the excreta as well as that of the feed were determined according to [34]. The digestibility of any nutrient was calculated using (input-output/input) × 100. 

Five animals were taken randomly from each treatment group to represent all treatment replications and slaughtered according to the Islamic method after being fasted overnight. The carcass and inner organs were separated, weighed, and expressed as relative to live BWG as cited by [9]. The empty gizzard and intestinal were used to estimate gizzard and intestinal percentage. 

At 35 days of age, five blood samples (5 mL per sample) of each treatment were collected in clean non-heparinized tubes. The serum was separated by centrifugation at 1500× *g* for 10 min at 4 °C, and stored at −18 °C until analysis. The serum profiles were determined using commercial diagnostic kits (Diamond Diagnostics Company, Cairo, Egypt). The serum total protein and albumin concentrations (g/dL) was established, according to Henry et al. [37] and Doumas et al. [38], respectively. The globulin concentrations (g/dL) were calculated as the difference between total protein and albumin. The activities (µ/L) of the alanine aminotransferase (ALT) and aspartate aminotransferase (AST) enzymes were determined according to the method described by Reitman and Frankel [39]. Serum creatinine and urea were determined by [40]. Total cholesterol was determined, as defined by Watson [41].

### 2.4. Statistical Analysis

Statistical analyses were performed using the GLM procedure of the statistical analysis software of the SAS Institute [42] using one-way ANOVA to test for the effects of seven treatments. In addition, a contrast analysis was used after exclusion of the control group to compare intermittent vs. continuous treatment. The linear and non-linear effects of enzymes dose were tested. In addition, a factorial analysis was run after excluding the control-unsupplemented treatment to check the result of enzyme level, administration method, and the interaction. Before analysis, all percentages data of digestibility measurements, carcass traits, and inner organs were transformed to arcsines to normalize data distribution. The pens were the experimental units. The mean differences at *p* ≤ 0.05 were tested using the Student–Newman–Keuls test. The *p* value between 0.05 and 0.10 was considered a trend. 

## 3. Results

It should be noted that results are presented according to the effect of contrasting intermittent vs. continuous methods of application, and then to the effect of treatments effects.

### 3.1. Growth, Feed Intake, and Survival Rate

The impact of administering multienzymes in drinking water on the growth and survival of broiler chickens are summarized in Table 2. Differences between (intermittent vs. continuous) were not significant for growth, FCR, and EPI during the experimental periods. 

Treatments had no significant effects on BWG during 1–21 days (*p* < 0.143) days of age but the effect approached meaningful (*p* < 0.099) during 22–35, and (*p* < 0.06) during the whole period showing the highest growth of groups on 1.5 mL/L drinking water administrated either intermittently or continuously. 

Regardless of administration method, enzyme level showed a significant effect on the growth of broilers during days 1–21 of age showing that groups supplemented with 1 mL/L enzymes increased growth compared to the group on 0.5 mL/L water. During days 22–35 of age, chicken that received the level of enzymes at 1.5 mL/L exhibited numerically (*p* < 0.061) higher BWG than those only given levels of 0.5 and 1 mL/L. For the whole period, 1.5 mL level of enzymes resulted in the growth of broilers more than 0.5 mL/L water. 

There no significant effect of administration method and the interaction between enzyme level and administration method on the growth of broilers during different periods. 

Feed intake of broiler chickens during most of the experimental periods was significantly affected by the administration method (intermittent vs. continuous), except for days 22–35 of age in which broilers in the intermittent group consumed significantly more feed than the continuous one. Treatments also had no significant impact on feed intake during the different experimental period, nor there were significant effects due to the enzyme level and the interaction between enzyme level and administration method. There were no deaths in this experiment; the survival rate was 100% during the experimental period, and thus death-rate data are not presented. 

### 3.2. Feed Conversion Ratio and European Production Index

The effects of administering multienzymes in drinking water on the FCR and EPI of broiler chickens are summarized in Table 3. The FCR and EPI were not significantly affected by the administration method (intermittent vs. continuous) during the experimental period. In addition, treatments did not affect FCR during most of the trial period except that FCR during days 1–35. In addition, the difference within 0.5, 1, and 1.5 mL levels was not significant between the two application methods. The results revealed that the group supplemented continuously with 1.5 mL/L water either continuously or intermittently show the best FCR, while the worst FCR was from a group that was supplemented with 0.5 mL/L intermittently. 

Irrespective of administration method, enzyme level significantly affect FCR during days 1–21 and FCR and EPI during days 1–35 of age, showing 1.5 mL/L water enhanced FCR and EPI compared to 0.5 mL/L water. The EPI was not significantly affected by administration method (intermittent vs. continuous) as well as treatments.

### 3.3. Apparent Digestibility of Nutrients

Data concerning the effects of the administration method and different enzyme treatments on the apparent digestibility of the nutrients of broiler chicks are shown in Table 4. There were no significant effects for the administration method (intermittent vs. continuous) and for the treatments on the apparent digestibility of nutrients. The results showed that there was a trend (*p* ≤ 0.11) for higher crude fiber digestibility of the group supplemented with enzymes continuously compared to that on intermittent supplementation. A trend towards increasing digestibility of crude protein (*p* ≤ 0.10), ether extracts (*p* ≤ 0.11), and ash (*p* ≤ 0.07) was shown because of different enzyme treatments compared to the control.

### 3.4. Carcass Traits and Inner Body Organs

The carcass characteristics and body organs of broilers as affected by the method of administration and different enzyme treatments are shown in Table 5. The percentages of the liver, heart, pancreas, abdominal fat, spleen, bursa, and thymus were not significantly affected by method of administration (intermittent vs. continuous). However, there was a trend (*p* < 0.09) of increased dressing (%) because of constant supplementation compared to the intermittent one, but reduced gizzard (*p* < 0.089) and pancreas (*p* < 0.059) percentage. There were no significant differences in the carcass traits and body organs due to different enzyme treatments.

### 3.5. Lymphoid Organs

The lymphoid organs of broilers as affected the method of administration (intermittent vs. continuous) and different enzyme treatments are shown in Table 6. The percentages of the spleen, bursa, and thymus were not significantly affected by method of administration (intermittent vs. continuous). There were no significant differences in the lymphoid organs due to different enzyme treatments.

### 3.6. Blood Serum Biochemical Constituents

There was no significant effect of the administration method (intermittent vs. continuous) on albumin, globulin, albumin/globulin ratio, total cholesterol, but a trend towards (*p* < 0.075) increasing serum protein was shown in the continuous group compared to intermittent group Table 7. 

### 3.7. Markers of the Liver and Renal Functions

Results for liver and renal functions of the broiler chicks as they were influenced by the administration method (intermittent vs. continuous) and different enzyme treatments are shown in Table 8. There was no significant effect of the administration mehtod (intermittent vs. continuous) on most of the indices of the liver and renal functions except for serum AST (*p* < 0.079) and Urea (*p* < 0.044). Both were higher in the group given enzymes continuously than those of the group supplemented with enzymes intermittently. There was no significant effect of different enzyme treatments on the markers of the liver and renal functions of the broiler chicks.

## 4. Discussion

The use of enzymes in feed to overcome the anti-nutritional factors, to increase feed utilization, and to improve performance as well as to decrease stress has received considerable attention [3,4,8,11,16,43]; so, also is the use of enzymes in water [26,27,28,29], with water application inducing superior performance [24]. This advantage may be due to faster distribution, application, and availability [24,25] and greater use of water as well [27]. However, the effect of the enzymes depends on diet composition (target substrate), the dose of the enzyme, and the age of chickens [5,9]. However, the activity of enzymes was not determined herein by direct enzymatic method, but by growth performance and digestibility of diets instead, showing some positive effects depend on the age of broilers, and dose of enzymes.

The use of enzymes is also restricted by its cost-benefit ratio [44]; hence, we investigated the effect of different doses of multienzymes continuously (24 h/d) or intermittently (12 h/d) administered with the aim of improving the performance of the animals and reducing the costs of supplementation on broiler performance and the EPI. The results indicate that at 1 and 1.5 mL of enzyme supplementation either continuously or intermittently yields superior effects on biological and economic value than the low dose of enzymes with a low dosage and intermittent administration could yield better economic benefits under similar production performance due to low cost of supplementation. 

The results indicate that the effect of the method of administration (intermittent vs. continuous) and different enzyme treatments are dependent on the age of the chickens (*p* < 0.034). It was observed that the level of enzyme supplemented at 1 mL/L water improved growth (7.8%) of chickens during only days 1–21 of age compared to 0.5 mL/L supplementation. During days 22–35 and 1–35 of age, enzyme level at 1.5 mL resulted in higher growth of broilers by 5.9% and 5.1%, respectively, compared to 0.5 mL. This indicates that enzymes supplemented continuously at 1.5 mL/L water of a corn-soybean diet at 22–35 days of age was adequate to improve growth and FCR. On the other hand, the dose of multienzymes given intermittently to induce similar improvements in FCR was 1 mL/L. Either continuous or intermittent supplementation with 1.5 mL/L water was more efficient for increasing growth (8.6%–9.9%) and improving FCR (7.7%) and EPI (19.3%) for the whole experimental period compared to unsupplemented control even not significantly different from the 1 mL multienzyme treatments, but obviously economically beneficial. Similar results were reported previously [17,45]. The linear and non-linear components of enzymes concentration showed a weak linear effect on BWG during 1–21 days of age (*p* < 0.089) and FCR during 1–21 days of (*p* = 0.076). In the literature, various cereals responded differently to enzyme supplementation with a greater effect on viscous grains such as rye [9,18,23]. These results indicate that the enhancing effect of increasing dose of enzymes on growth and FCR of broilers occurs during the 1–21 days of age period. 

Multienzymes increased the digestibility of CP, EE, and CF and ash numerically of the corn-soybean diet containing 5% rye, and the saturation response was attained in the group supplemented with 1 mL/L water due to lack of response to further increase in enzyme concentration. This could explain the positive effect of enzymes on the performance of broilers found herein. The positive response of the corn-soybean meal diet to multienzymes indicates the presence of insoluble components, such as NSP in rye, which are not digested by broiler chickens and may limit the utilization of some nutrients or negatively influence gut health, nutrient digestibility, and dietary metabolizable energy [4,7,43,46]. The results cited herein are similar to those reported by [8,20,43] and can be explained by the improvement in gut architecture and health [4,6,47].

Dressing percentage increased (1.6%) due to enzyme supplementation continuously in comparison to the intermittent method, showing the positive effect of continuous enzyme supply on the availability of nutrients. It seems that increasing nutrient availability due to continuous supplementation will be used for tissue growth. 

The results of this study indicate that enzyme supplementation through different methods at different doses did not affect lymphoid organs (spleen, bursa, and thymus) of broilers. There was an increase in serum protein and albumin (no specific immune protein) due to enzyme supplementation when continuously administered; this may be a reflection of the increase in digestibility of CP observed herein. This positive relationship between enzymes and immunity was recently reported [48,49]. Further evidence indicates a lack of adverse effects of enzymes on the markers for liver leakage and renal function, as enzyme supplementation in water significantly decreased AST and ALT, showing a decrease in liver leakage enzymes, showing an improvement in liver function [26,27,28,29].

## 5. Conclusions

The highest BWG and the best FCR and PI for the whole experimental period were from broilers given continuous or intermittent multienzyme supplementation at 1.5 mL/L drinking water suggesting further experiments should be undertaken to test the possibility of constant vs. intermittent application of multienzymes due to 50% saving in the cost of enzyme supplementation. In addition, intermittent supplementation of enzymes at 1 mL/L drinking water and continuous supplementation at 0.5 mL/L drinking water can be investigated in further experiments as a tool to improve broiler performance and European production index. 

## Figures and Tables

**Table 1:** The ingredients and determined and calculated composition of the experimental diets.

Ingredients	Diets, % as Fed Basis	
	Starter (1–21 d of Age)	Grower (22–35 d of Age)
Maize	51.3	51.9
Rye	0.0	5.0
Soybean meal (44% Crude protein)	32.8	24.4
Vegetable oil	2.25	2.0
Full fat soybean meal	10.0	13.0
Dicalcium phosphate	1.8	1.6
Limestone	1.0	1.0
L-Lysine	0.10	0.15
DL-Methionine	0.15	0.2
Vitamins and minerals premix *	0.3	0.3
NaCl	0.3	0.45
Calculated ^1^ and determined ^2^ composition (% as fed basis)		
Dry matter ^2^	89.15	88.85
Metabolizable energy (MJ/kg) ^1^	12.73	12.98
Crude protein ^2^	22.8	21.2
Total lysine ^1^	1.33	1.23
Total methionine ^1^	0.50	0.52
Total methionine+cysteine ^1^	0.87	0.87
Ash ^2^	5.41	5.76
Calcium ^1^	0.91	0.85
Available phosphorus ^1^	0.46	0.41
Ether extract ^2^	6.42	6.68
Crude fiber ^2^	3.55	4.48

* Vitamins and minerals mix. provides per kg diet: Vit. A, 12,000 IU, vit. E (dl-α-tocopheryl acetate) 20 mg, menadione 2.3 mg, Vit. D3, 2200 ICU, riboflavin 5.5 mg, calcium pantothenate 12 mg, nicotinic acid 50 mg, Choline 250 mg, vit. B12 10 μg, vit. B6 3 mg, thiamine 3 mg, folic acid 1 mg, d-biotin 0.05 mg. Trace mineral (mg/kg of diet): Mn 80 Zn 60, Fe 35, Cu 8, Selenium 0.1 mg. ^1^ Calculated,^2^ Determined.

**Table 2:** Effects of multienzymes supplemented continuously or intermittently on body weight gain and feed intake of Arbor Acres broiler chicks during 1–35 days of age.

Treatments	Body Weight Gain, g/Chick/Period/Days			Feed Intake, g/Chick/Period/Days		
	1–21	22–35	1–35	1–21	22–35	1–35
**Administration Method**						
Int	609	1051	1660	957	1839 ^a^	2795
Con	615	1067	1682	954	1832 ^b^	2787
Enzyme level						
0.5	590 ^b^	1038	1627 ^b^	957	1835	2792
1.0	636 ^a^	1041	1677 ^ab^	955	1827	2792
1.5	611 ^ab^	1099	1710 ^a^	955	1833	2788
Treatments						
Control	577	991	1568	958	1839	2797
0.5 × Int	568	1027	1595	956	1837	2793
0.5 × Con	611	1048	1659	957	1835	2792
1.0 × Int	637	1032	1669	954	1837	2791
1.0 × Con	634	1051	1685	956	1837	2793
1.5 × Int	621	1096	1717	960	1838	2798
1.5 × Con	600	1103	1703	949	1830	2779
SEM	20.2	27.7	36.4	4.29	2.22	4.79
*p* value						
Contrast Int vs. Con	0.147	0.589	0.225	0.896	0.048	0.770
Treatments	0.143	0.099	0.060	0.717	0134	0.174
Enzyme level	0.047	0.061	0.046	0.919	0.392	0.720
Administration method	0.669	0.489	0.402	0.542	0.038	0.141
Interaction	0.197	0.963	0.473	0.351	0.352	0.129

a,b: Differences among means within a column within each factor not sharing similar superscripts are significant at *p* < 0.05; Int: intermittent; Con: continuous; SEM: standard error of the mean.

**Table 3:** Effects of multienzyme supplemented continuously or intermittently on feed conversion ratio and European production index of Arbor Acres broiler chicks during 1–35 days of age.

Treatments	Feed Conversion kg/kg/Period/Days			EPI
	1–21	22–35	1–35	
**Administration Method**				
Int	1.58	1.75	1.69	290
Con	1.56	1.72	1.66	299
Enzyme level				
0.5	1.64 ^a^	1.77	1.72 ^a^	279 ^b^
1.0	1.51 ^ab^	1.77	1.67 ^ab^	296 ^ab^
1.5	1.57 ^b^	1.67	1.63 ^b^	308 ^a^
Treatments				
Control	1.68	1.86	1.79 ^a^	260
0.5 × Int	1.70	1.79	1.76 ^a^	268
0.5 × Con	1.57	1.75	1.69 ^ab^	290
1.0 × Int	1.50	1.79	1.67 ^ab^	293
1.0 × Con	1.51	1.76	1.66 ^b^	299
1.5 × Int	1.55	1.68	1.63 ^b^	309
1.5 × Con	1.58	1.66	1.63 ^b^	307
SEM	0.056	0.046	0.039	12.5
*p* Value				
Contrast Int vs. Con	0.112	0.570	0.228	0.229
Treatments	0.115	0.075	0.047	0.065
Enzyme level	0.039	0.053	0.036	0.049
Administration method	0.474	0.438	0.361	0.359
Interaction	0.188	0.975	0.515	0.558

a,b: Differences among means within a column within each factor not sharing similar superscripts are significant at *p* < 0.05; EPI: European production index; Int: intermittent; Con: continuous; SEM: standard error of the mean.

**Table 4:** Effects of multienzymes supplemented continuously or intermittently on apparent nutrient digestibility (%) during 36–40 days of age of Arbor Acres broiler chicks.

Treatments	Apparent Digestibility, %				Ash%
	Dry Matter	Crude Protein	Ether Extract	Crude Fiber	
**Administration Method**					
Int	75.1	80.1	85.2	21.8	37.8
Con	75.8	80.9	86.1	23.8	39.0
Treatments					
Control	72.3	73.8	77.9	13.2	33.5
0.5 × Int	74.7	76.3	81.2	20.0	40.1
0.5 × Con	77.4	79.4	84.5	20.0	36.1
1.0 × Int	75.2	79.8	84.9	21.0	33.8
1.0 × Con	74.6	82.5	87.8	25.5	40.9
1.5 × Int	75.5	84.1	89.4	24.3	39.5
1.5 × Con	75.3	80.9	86.1	26.0	39.9
SEM	1.19	1.53	1.63	1.46	2.18
*p* value					
Contrast Int vs. Con	0.510	0.510	0.500	0.110	0.510
Treatments	0.330	0.100	0.110	0.320	0.070

Int: intermittent; Con: continuous; SEM: standard error of the mean.

**Table 5:** Effects of multienzymes supplemented continuously or intermittently on carcass and organs characteristics of Arbor Acres broiler chicks during 1–35 days of age.

Treatments	Relative Weight (%)						
	Dressing	Gizzard	Liver	Heart	Intestinal	Pancreas	Abdominal Fat
Administration method							
Int	72.9	1.77	2.71	0.672	3.90	0.374	0.994
Con	74.1	1.61	2.84	0.693	4.60	0.331	1.18
Treatments							
Control	69.6	1.90	3.21	0.735	5.98	0.390	1.48
0.5 × Int	72.1	1.86	2.75	0.678	4.46	0.398	1.18
0.5 × Con	73.3	1.69	3.03	0.680	5.54	0.312	1.36
1.0 × Int	72.8	1.82	2.86	0.646	3.66	0.376	0.877
1.0 × Con	74.5	1.63	2.99	0.707	4.09	0.352	1.07
1.5 × Int	73.9	1.63	2.52	0.692	3.58	0.347	0.921
1.5 × Con	74.5	1.52	2.50	0.691	4.16	0.329	1.11
SEM	0.82	0.113	0.200	0.052	0.421	0.027	0.170
*p* value							
Contrast Int vs. Con	0.090	0.089	0.445	0.617	0.471	0.059	0.185
Treatments	0.822	0.950	0.757	0.795	0.714	0.396	0.999

Int: intermittent; Con: continuous; SEM: standard error of the mean.

**Table 6:** Effects of multienzymes supplemented continuously or intermittently on lymphoid organs of Arbor Acres broiler chicks during 1–35 days of age.

Treatments	Relative Weight, %		
	Spleen	Bursa	Thymus
Administration method			
Int	0.157	0.295	0.551
Con	0.153	0.274	0.517
Treatments			
Control	0.172	0.288	0.709
0.5 × Int	0.149	0.283	0.572
0.5 × Con	0.145	0.289	0.560
1.0 × Int	0.161	0.279	0.518
1.0 × Con	0.163	0.273	0.398
1.5 × Int	0.162	0.321	0.562
1.5 × Con	0.152	0.260	0.591
SEM	0.003	0.006	0.034
*p* value			
ContrastInt vs. Con	0.777	0.341	0.496
Treatments	0.939	0.397	0.460

Int: intermittent; Con: continuous; SEM: standard error of the mean.

**Table 7:** Effects of multienzymes supplemented continuously or intermittently on biochemical constituents of blood serum of Arbor Acres broiler chicks during 1–35 days of age.

Treatments	Biochemical Constituents of Blood Serum				
	Protein (g/dL)	Albumin (g/dL)	Globulin (g/dL)	Albumin/Globulin Ratio	Total Cholesterol (mg/dL)
Administration method					
Int	5.44	2.87	2.57	1.29	187
Con	6.28	3.46	2.82	1.61	195
Treatments					
Control	4.36	2.54	1.82	1.60	192
0.5 × Int	5.11	2.70	2.41	1.34	174
0.5 × Con	5.74	3.44	2.30	1.64	189
1.0 × Int	5.94	3.04	2.90	1.16	199
1.0 × Con	6.71	3.44	3.27	1.20	191
1.5 × Int	5.26	2.86	2.40	1.36	188
1.5 × Con	6.41	3.50	2.91	1.98	205
SEM	0.562	0.275	0.511	0.456	10.49
*p* value					
Contrast					
Int vs. Con	0.075	0.131	0.546	0.397	0.355
Treatments	0.894	0.819	0.821	0.817	0.426

Int: intermittent; Con: continuous; SEM: standard error of the mean.

**Table 8:** Effects of multienzymes supplemented continuously or intermittently on liver function and renal function of Arbor Acres broiler chicks during 1–35 days of age.

Treatments	Liver Function			Renal Function		
	ALT, u/L	AST, u/L	ALT/AST	Urea, g/dL	Creatinine, g/dL	Urea/Creatinine Ratio
**Administration Method**						
Int	48.1	59.5	1.23	23.6	1.21	19.9
Con	50.0	62.3	1.26	26.6	1.26	21.5
Treatments						
Control	52.0	60.0	1.16	25.5	1.28	20.2
0.5 × Int	47.6	58.4	1.23	22.2	1.27	18.4
0.5 × Con	52.0	62.4	1.21	29.0	1.24	23.8
1.0 × Int	49.2	64.0	1.30	25.0	1.16	21.6
1.0 × Con	48.0	64.8	1.36	26.6	1.25	21.5
1.5 × Int	47.6	56.0	1.18	23.5	1.21	19.6
1.5 × Con	50.0	59.6	1.2	24.2	1.28	19.3
SEM	1.56	1.88	0.047	1.80	0.094	1.75
*p* value						
Contrast Int vs. Cont	0.153	0.079	0.603	0.044	0.570	0.278
Treatments	0.208	0.654	0.717	0.195	0.816	0.219

Int: intermittent; Con: continuous; ALT: alanine aminotransferase; AST: aspartate aminotransferase; SEM: standard error of the mean.
